# Accelerating development, registration and access to medicines for rare diseases in the European Union through adaptive approaches: features and perspectives

**DOI:** 10.1186/1750-1172-9-20

**Published:** 2014-02-10

**Authors:** David Uguen, Thomas Lönngren, Yann Le Cam, Sarah Garner, Emmanuelle Voisin, Carlo Incerti, Marc Dunoyer, Moncef Slaoui

**Affiliations:** 1 Voisin Consulting Life Sciences, Paris, France; 2 NDA Advisory Services Ltd, Leatherhead, Surrey, UK; 3 European Organisation for Rare Diseases (EURORDIS), Paris, France; 4 Centre for the Advancement of Sustainable Medical Innovation, London, UK; 5 Genzyme Corporation, Cambridge, USA; 6 GSK London, London, UK

**Keywords:** Orphan medicinal products, Rare diseases, Adaptive licensing, Patient access

## Abstract

There is growing recognition that the current research-and-development (R&D) and innovation-regulation ecosystem could be made more efficient to stimulate and support access to innovative therapies for those patients with rare, life-threatening diseases for which there are no adequate licensed therapies. New and progressive thinking on the principles and processes of drug development and regulation are needed in rare disease settings in order to ensure developments are financially sustainable. This paper presents perspectives on the current and emerging schemes for accelerating development of and access to medicines for rare diseases in the European Union.

## Introduction

Rare diseases represent a key challenge to healthcare systems. With less than 2% of identified rare diseases currently covered by approved treatments, the rare diseases population is underserved. There is a need to increase the number of rare diseases treated and to achieve this end in a financially sustainable manner. Regulatory authorities, patient groups, pharmaceutical companies, legislators and payers collectively face the dilemma of how best to help achieve more timely access to new effective treatments for patients with chronically debilitating and life-limiting conditions. Collaborative dialogue between all stakeholders is essential and is already identifying issues and informing the debate on a number of topics. Issues include how to adapt clinical trial and research mechanisms to suit rare disease settings while retaining the necessary rigors of evidence-based medicine. There needs to be debate on how post-authorisation evidence-gathering and collection of non-randomised trial evidence might help expedite access to orphan medicinal products (OMPs). An additional issue is how to sustain investment in innovative research while ensuring access can be afforded by healthcare systems. Improving access to OMPs should not come at an inflated cost – patients, payers and drug developers should each derive value from genuine therapeutic advances [1]. These topics are broader than the regulatory debate on adaptive licensing that is being piloted and championed by the EMA, and extend beyond the market access sentinel of health technology appraisals. The very rare and serious nature of the clinical conditions in which OMPs are investigated affects not only the type of clinical research that can be undertaken, but also typically influences the patient perspective on the benefit:risk profile of a health technology. This paper provides a contemporary overview of how rare diseases and OMPs challenge purely gatekeeper-concepts of regulation and market access, by embracing multi-stakeholder views and taking a dynamic and fresh look at how best to integrate knowledge, evidence and experience in order to meet rare disease needs.

## Licensing mechanisms

There are two European mechanisms by which an orphan medicinal product (OMP) can currently be fast-tracked through the licensing system - these are registration under-exceptional-circumstances (EC) and the granting of a conditional-marketing authorization (CA). In brief, EC is applicable when there appears to be a positive benefit:risk balance for a drug but comprehensive efficacy and safety data cannot be provided even in the long-term (for reasons to do with the rarity or complexity of the disease). A CA is issued on condition that the applicant will provide missing data within an agreed timeframe after approval.

While OMP designation incentivizes drug development for rare conditions, the rate of OMPs products reaching the market has remained frustratingly flat [2]. Out of the 70 OMPs in Europe in the past decade, only 15 have received exceptional circumstances (EC) authorization and 5 have been granted CA. This reflects the fact that many OMPs do not or cannot meet the current criteria (see above) for EC or CA.

There is a compelling case for exploring how the regulatory processes and healthcare systems can be used to support research and development whilst collecting the data that is necessary to confirm the clinical profile of a product and thus support patient access to OMPs that offer acceptable safety and effectiveness. Adaptive licensing is one of a number of approaches currently proposed [3].

## Features, rationale and potential impact of adaptive licensing initiatives

The EMA’s ‘Road map to 2015’ states an intention to explore how the process that are run under the existing regulatory legislation could be adapted to support innovation [4]. The rationale for adaptive approaches to licensing is to allow certain new therapies to be appraised on the basis of an initial positive risk:benefit in a small group of patients and to later confirm and broaden this appraisal by using additional clinical data obtained post approval from, for example, registries. This is then reinforced by prospectively planned, adaptive and iterative phases of evidence-gathering [3]. Adaptive licensing differs from traditional dichotomous license decisions where the presumption is that an experimental therapy, on receiving a license, is safe and efficacious for use in a given patient population [3-5]. The adaptive process would involve a graded, tightly-managed market entry, to account for higher risk and higher uncertainties than that involved in the conventional licensing paradigm. Although effected through a different model, the goals and principles of adaptive licensing are in tune with the reality of current drug evaluation paradigms, which themselves involve, post-licence, a continuum of active surveillance, additional studies, and capture and assessment of real-life data. Patients with very rare and very serious diseases for which there are no currently available therapies, might be expected to be more prepared than other patient groups to accept the potential risks of having earlier access to therapies while these are still being evaluated. We believe that some OMPs, aimed at treating certain rare conditions may be well suited to the model provided by adaptive licensing.

Proponents of adaptive licensing view it as an evolutionary step, extending elements of existing clinical development and licensing programmes, and believe that such an approach is vital to support and sustain the continued development and access to new and innovative medicines. However concerns have been raised that such a scheme would require a radical transformation in the regulatory framework or would serve as a conduit for premature drug approvals on commercial rather than clinical grounds.

## Patients’ perspective

Representatives of patients with rare diseases support moves that would allow quicker access to safe and efficacious medicines but highlight the need for a pan-European approach, before and after marketing authorization, to ensure that access, founded on clinical evaluation, is not fragmented at national level on grounds of cost considerations taking precedence over clinical value and utility. Those with rare diseases are aware that while the ideal is to seek a drug license armed with a comprehensive clinical data dossier, the reality of rare diseases requires clinical study in small and heterogeneous patient populations, and must rely heavily on real-life studies, registry reports and compassionate-use programmes to prospectively generate sufficient data for meaningful evaluation of drug efficacy and safety.

## Regulator’s perspective

In considering the development of new treatments using an adaptive model, the role of regulators changes from ‘gate-keeper’ to enabler. This will require open dialogue with clinical experts, payers, industry and patients to provide the necessary environment, infrastructure and investment in anticipation of the confirmation of a product’s effectiveness. Collaborative and horizon-scanning approaches will allow developing more innovative development programmes and trial designs incorporating the most appropriate endpoints, patient-reported outcomes and quality of life measures by which to guide and gauge treatment success. The existing mechanisms for “scientific advice” are currently evolving to cover this enlarged scope.

## Policy-makers’ perspective

Policy makers also appreciate that in rare disease settings, therapeutic innovation is not always successfully translated into patient benefits. The feasibility of flexible and innovative clinical trial designs (including adaptive study designs), and the timeframes for post-approval obligations need to be collaboratively explored upfront. The concept of adaptive licensing is seen as pragmatic and encourages drug assessment and approval as a continuum. In this continuum, access to a therapeutic innovation may be possible earlier and the type of evidence submitted in support of access to a novel therapy may differ from traditional approval and appraisal routes. Organizations such as NICE are already open to the submission of non-randomized controlled trial evidence providing the inherent biases have been adequately explored. There are also decision options that allow for payment whilst the necessary confirmatory evidence is being collected. It will be important however to ensure that any time-efficiency gains are included in cost considerations which need to encompass all development costs in addition to drug pricing and cost-effectiveness evaluations.

## Benefits, challenges and points of debate

Rare diseases represent uncharted territory and rarity makes knowledge hard to obtain. In some instances, rare diseases and their OMPs meet the criteria for EC and CA and innovative products can be made available to patients through these routes. However, often the patient characteristics and the risk:benefit balance at stake in rare conditions require a different paradigm and mechanisms. The concept of adaptive licensing allows default to a progressive regulatory assessment and quicker coordinated access for OMPs. An additional benefit is greater engagement of healthcare providers with the R&D process.

Potential challenges relate to managing the trade-off between evidence-generation and access and in particular the need to reconcile and develop a robust evidence-collection system. Other challenges include determining which specific OMPs and rare diseases might be eligible for pilot schemes such as the EMA adaptive licensing process, and devising mechanisms for deciding which route and method of evidence collection best suits which OMP and its disease setting. Flexible and innovative pricing mechanisms based on real-life value assessment and realized by contractual agreements could also address country-specific issues of affordability.

Much needs to be done to move from the current conceptual vision for adaptive licensing. This will require political and cultural changes in thinking, such that all stakeholders understand the risks, share the responsibilities and make the investments necessary for the process to be developed.

Close and frequent dialogue, interactions between the sponsors, regulators, payers and patient groups should be encouraged and formalized. The rewards for embracing change and exploring new models will be found in providing innovative treatments for patients with unmet needs.

## Abbreviations

OMP: Orphan medicinal product; CA: Conditional authorization; EC: Authorization under exceptional circumstances; EMA: European medicines agency; NICE: National institute for health and clinical excellence.

## Competing interests

Monclef Slaoui and Marc Dunoyer are employed by GSK. Carlo Incerti is employed by Genzyme.

## Authors’ contributions

The article is based on presentations and panel-audience discussion during a 2-hour roundtable meeting held on March 1 2013 (Rare Disease Day 2013). The meeting was supported by GlaxoSmithKline (GSK). The meeting was chaired by Thomas Lönngren and presentations were made by David Uguen, Yann Le Cam and Sarah Garner. Monclef Slaoui, Emmanuelle Voisin, Marc Dunoyer and Carlo Incerti were panellists. A draft of this Letter was prepared by Livewire Communications UK, based on the roundtable meeting content, after which all authors validated the content and contributed to the draft. All authors have given final approval of the version to be published.

## Authors’ information

David Uguen works for Voisin Consulting Life Sciences, managing projects involving the design and implementation of regulatory strategies for the development, registration and maintenance of pharmaceutical and biotechnology medicinal products in Europe.

Thomas Lönngren is an independent strategic advisor to NDA Advisory Services Ltd, through his company Pharma Executive Consulting. Dr Lönngren was previously Executive Director of the EMA where he served his 10-year term from 2001–10.

Yann Le Cam is Chief Executive Officer and co-founder of EURODIS, and has worked for non-governmental organizations and in patient advocacy for 25 years. He is vice chairman of the EU Committee of Experts on Rare Diseases (EUCERD) and has served as a patient representative to the EMA Committee for Orphan Medicinal Products (COMP).

Sarah Garner is Associate Director for Research and Development at the National Institute for Health and Care Excellence (NICE) and project Director for Adaptive Licensing at the UK Centre for the Advancement of Sustainable Medical Innovation (CASMI).

Moncef Slaoui is Chairman of Global Research and Development (R&D) and Vaccines, R&D Management, at GSK.

Emmanuelle Voisin is the Principal and founder of Voisin Consulting Life Sciences, Paris, France.

Marc Dunoyer is Global Head of GSK’s Rare Diseases Unit.

Carlo Incerti leads Genzyme’s Global Medical Research and Development function.
